# Intravascular Papillary Endothelial Hyperplasia of the Upper Limb Mimicking a Vascular Neoplasm: Case Report and Literature Review

**DOI:** 10.1016/j.ejvsvf.2026.07.003

**Published:** 2026-07-13

**Authors:** María Gutiérrez Fernández de Velasco, Geraldinne Raiveliz Ruiz Quintero, Xabier Cabezuelo Adame, Melina Vega de Ceniga

**Affiliations:** a Department of Vascular Surgery, H. Galdakao Usansolo, Bizkaia, Spain; b Department of Pathology, H. Galdakao Usansolo, Bizkaia, Spain

**Keywords:** Intravascular papillary endothelial hyperplasia, Masson’s tumour, Upper extremity vascular lesions, Vascular surgery

## Abstract

**Introduction:**

Intravascular papillary endothelial hyperplasia (IPEH), also known as Masson’s tumour, is a rare benign vascular lesion caused by reactive endothelial proliferation within vascular spaces. Despite its benign nature, IPEH may clinically and radiologically mimic malignant vascular tumours, particularly when arising in the upper extremity, posing a diagnostic challenge.

**Report:**

A 38 year old woman presented with a slowly enlarging subcutaneous nodule in the left antecubital fossa, with no history of trauma. Ultrasonography revealed a well defined solid mass with heterogeneous echotexture and suspected vascular communication. Magnetic resonance angiography demonstrated a solid lesion in continuity with the ulnar artery, with segmental arterial interruption and distal reconstitution, raising concern for a vascular neoplasm. An *en bloc* surgical resection was performed, including ligation of the involved ulnar vessels, without compromising distal perfusion. Histopathological examination showed papillary endothelial proliferation confined within a venous type vessel, without cytological atypia or necrosis, confirming primary IPEH.

**Discussion:**

To contextualise this case, a review of the literature was conducted, identifying 103 reported cases of IPEH involving the upper extremities. Lesions most frequently affected the fingers, followed by the forearm and elbow, with a slight female predominance and a mean age in the fourth decade of life. Primary (type I) IPEH accounted for nearly all cases. Complete surgical excision was associated with excellent outcomes and low recurrence rates. Awareness of IPEH is important, and it should be included in the differential diagnosis of vascular tumours to avoid unnecessarily aggressive oncological resections, although complete surgical excision remains the standard initial management.

## Highlights

• A rare intravascular papillary endothelial hyperplasia affecting the ulnar artery.
• This case highlights the diagnostic pitfall mimicking vascular neoplasm.
• This review includes > 100 upper limb cases with clinical insights.
• Practical guidance is given on surgical management and recurrence prevention.
• This case provides educational value for vascular surgeons and pathologists.

## Introduction

Nodules in the hand are a common clinical finding and distinguishing between benign and malignant tumours is challenging, especially in rare vascular entities. Proper identification is essential to ensure appropriate management.

Intravascular papillary endothelial hyperplasia (IPEH), also known as Masson’s tumour, is a benign vascular lesion characterised by the papillary proliferation of endothelial cells within normal or dilated vascular spaces.^1^, ^2^, ^3^ First described by Dr Masson in 1923, the term IPEH was not used until 1976, when it was understood to be a reactive phenomenon rather than a true neoplasm.^4^^,^^5^

Three types of IPEH have been described in the literature.^2^ Primary IPEH (type I) arises within the lumen of a dilated vessel without associated vascular anomalies and represents the most common type.^5^ Secondary IPEH (type II) develops within pre-existing vascular lesions, such as haemangiomas or arteriovenous malformations. The rarest form is extravascular (type III), which arises within a haematoma.^6^^,^^7^

Clinically, it usually presents as a palpable soft tissue mass and may mimic more aggressive neoplasms, such as sarcomas, particularly on imaging studies.^1^, ^2^, ^3^

This report presents a case of IPEH in a 38 year old woman presenting with a nodular lesion on her left upper limb. In addition, it provides an updated review of the literature, including more than 100 reported cases of IPEH involving the upper extremities, focusing on epidemiological characteristics, anatomic distribution, clinical presentation, treatment strategies, and recurrence rates.

## Case report

A 38 year old woman presented with a nodular lesion of the left upper limb that had slowly enlarged over approximately one year. There was no history of trauma, and the lesion caused mild local discomfort.

Physical examination revealed a subcutaneous, fibroelastic nodule measuring 2.5 × 3 cm and located in the left antecubital fossa. The lesion was non-pulsatile, non-tender, and non-adherent to deep planes. Radial and ulnar pulses were present and symmetrical in both upper limbs.

**Figure 1:**
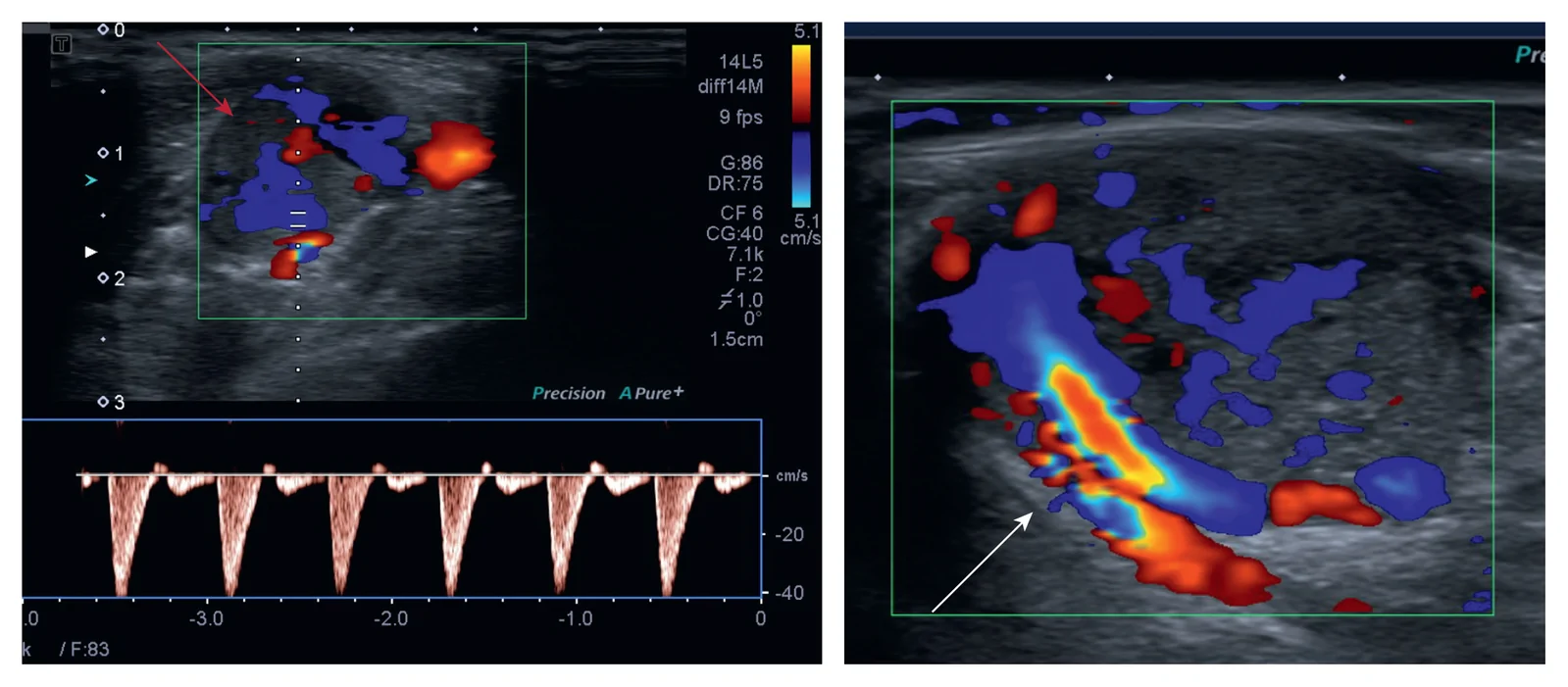
Ultrasound image showing a well defined, heterogeneous solid mass in the left antecubital fossa, with internal vascularity (red arrow) and an adjacent tubular structure suggestive of venous communication (white arrow).

Ultrasonography showed a 2.5 x 2 cm solid mass with well defined margins and heterogeneous echographic pattern, containing several anechoic areas. One of these areas showed tubular morphology suggestive of communication with a brachial vein (Fig. 1). The ulnar artery adjacent to the lesion was not clearly visualised as a patent tubular structure; however, distal flow was detected, suggesting reconstitution beyond the lesion.

**Figure 2:**
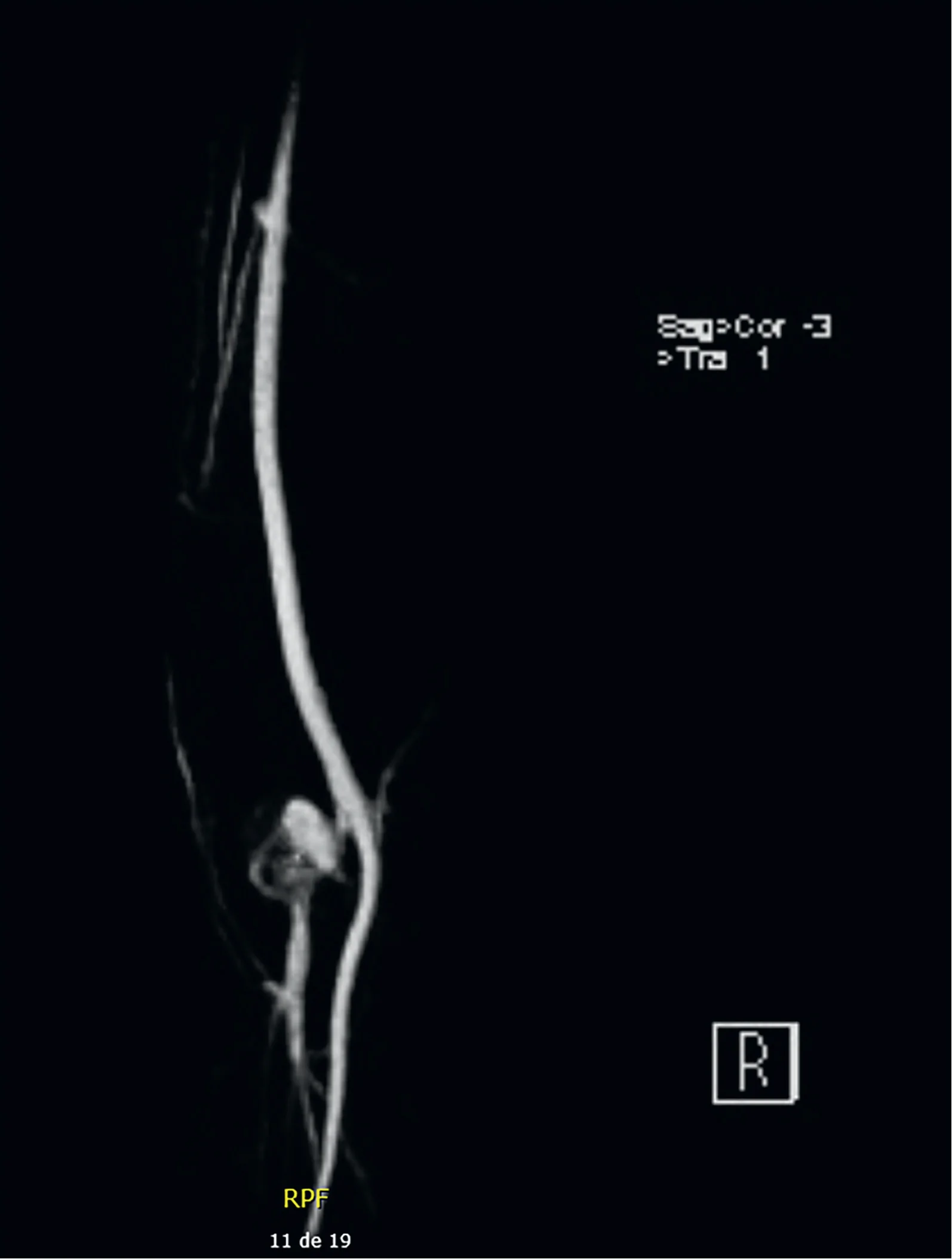
Contrast enhanced MR angiography with 3D reconstruction and maximum intensity projection (MIP), showing a well defined lesion in continuity with the proximal ulnar artery, with segmental interruption and distal reconstitution of flow.

Contrast enhanced magnetic resonance angiography with three dimensional reconstruction and maximum intensity projection revealed a solid lesion measuring 2.5 × 1.5 × 1.8 cm with well defined margins, appearing in continuity with the proximal ulnar artery. The artery was interrupted over a 1.5 cm segment adjacent to the lesion, with distal reconstitution of flow (Fig. 2). Although a venous malformation could not be definitively excluded, the presence of a solid component raised suspicion of a neoplasm with secondary vascular involvement.

A longitudinal incision over the antecubital fossa was used to expose the lesion and an *en bloc* resection was performed. The lesion involved the proximal ulnar artery and vein without extension to the brachial bifurcation. The affected ulnar artery and veins were ligated and divided. The specimen was sent in its entirety for definitive histopathological examination. Post-operatively, the patient remained asymptomatic, with preserved radial and ulnar pulses at the wrist and adequate hand perfusion.

**Figure 3:**
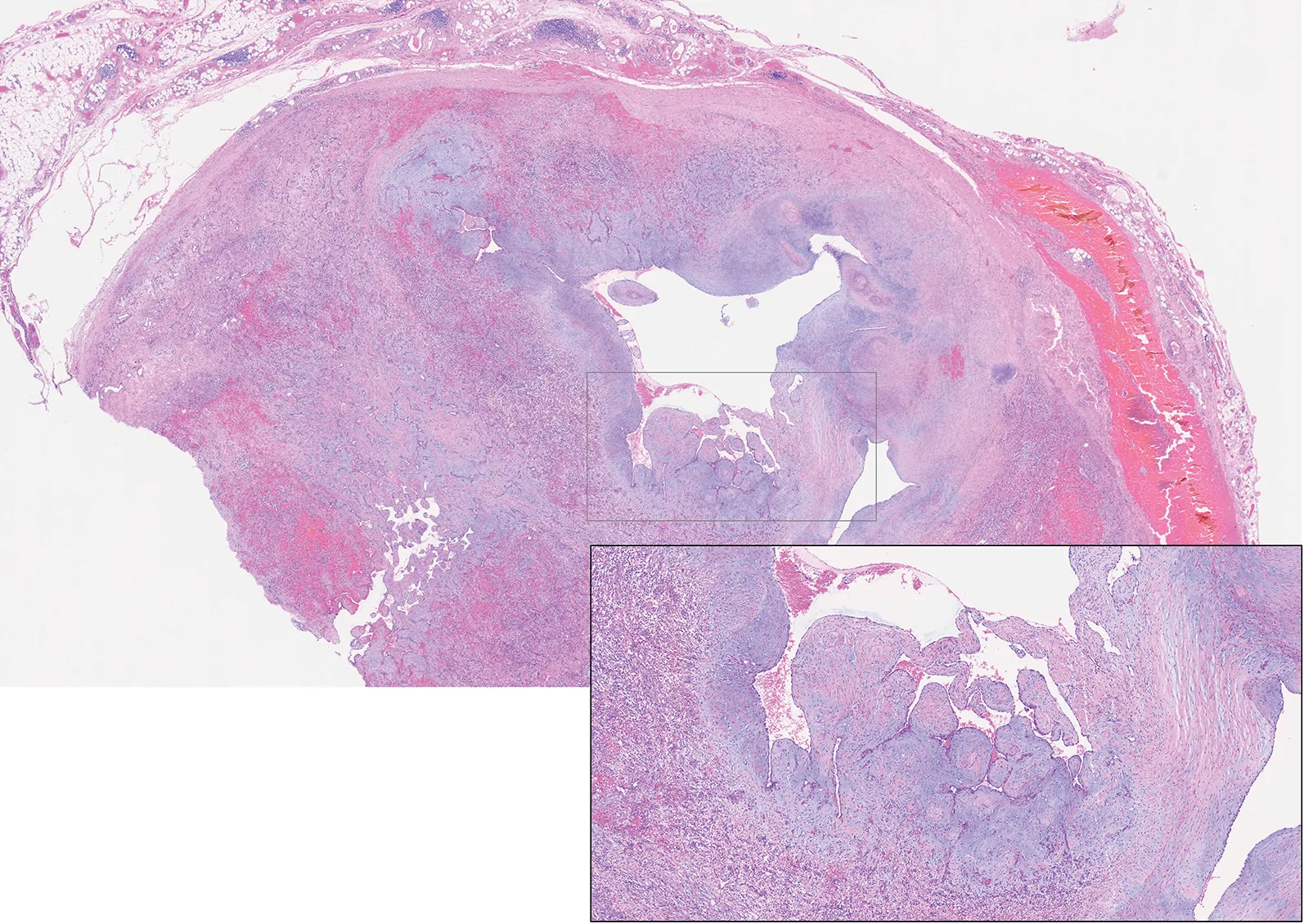
Haematoxylin and eosin stained histological section showing a well circumscribed intravascular papillary endothelial proliferation without cytological atypia or necrosis, consistent with intravascular papillary endothelial hyperplasia (Masson’s tumour).

Macroscopically, a 2.4 cm nodular formation with a red black solid soft surface was observed. Histopathological examination revealed a well circumscribed lesion arising within the wall of a venous type vessel, comprising papillary endothelial proliferation forming vascular channels of varying size. Occasional mitotic figures, lymphocytic infiltration, and areas of haemorrhage at different stages were observed. No necrosis or cytological atypia was present (Fig. 3). These findings were consistent with intravascular papillary endothelial hyperplasia.

The resection was complete and the patient remained asymptomatic with no evidence of recurrence at 12 month follow up; no additional treatment was required.

### Literature review

**Figure 4:**
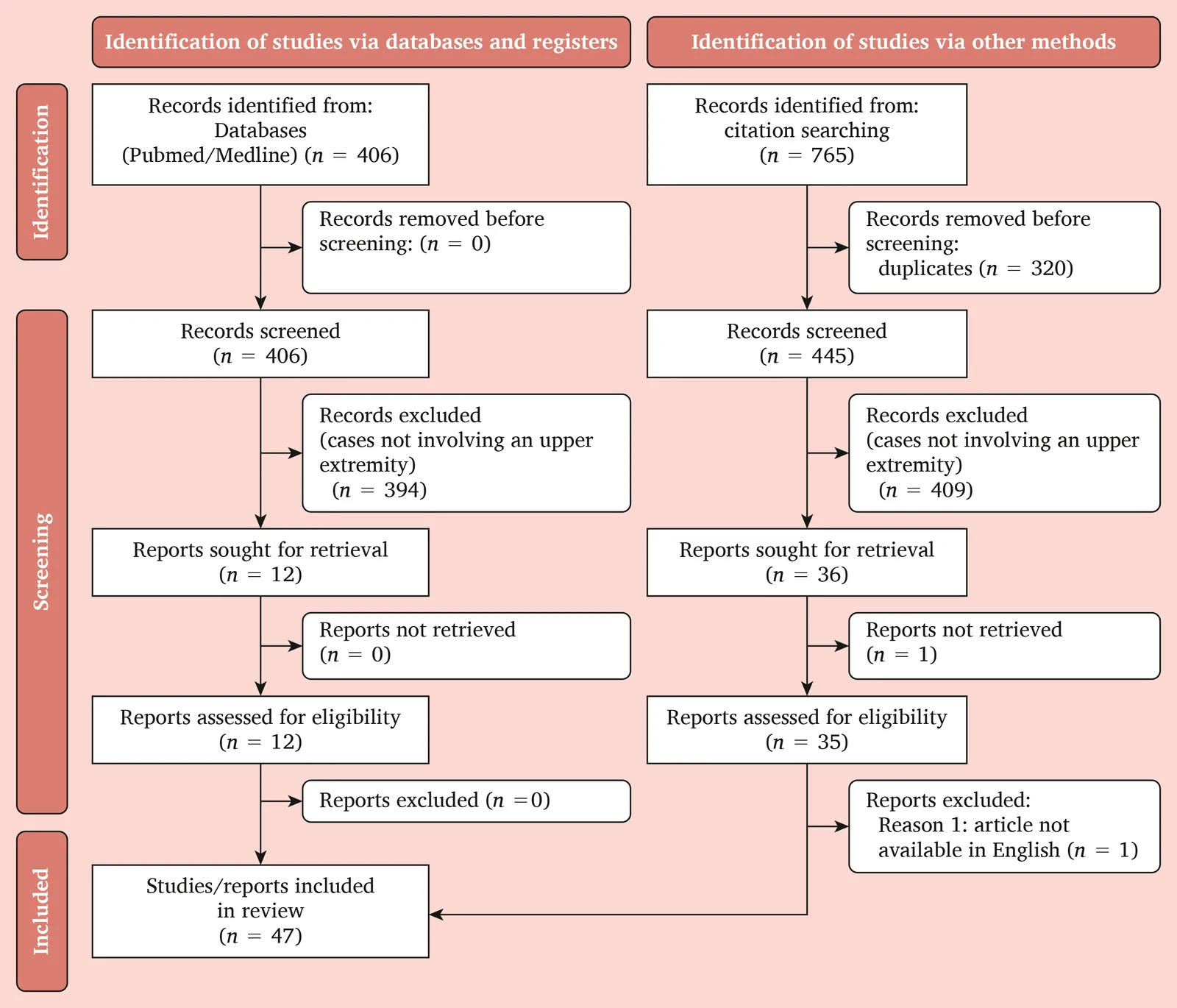
PRISMA 2020 flow diagram of the literature search and study selection process.

A PubMed and Medline search up to December 2024 was complemented by reference screening. Forty-seven published articles reporting cases of IPEH affecting the upper extremities were identified. The selection process followed PRISMA 2020 (Fig. 4). The main findings are summarised in Table 1, and the full list of included studies is provided in Appendix A.

**Table 1:** Summary of reported cases of intravascular papillary endothelial hyperplasia (IPEH) involving the upper extremities, including patient age and sex, lesion location and appearance, relevant medical history, clinical presentation, treatment, and recurrence.

Reference	Age – y	Sex	Location	History	Clinical presentation	Treatment	Recurrence
Hutcheson *et al.*, 2012^1^	63	Woman	Left thumb	NR	Decreased sensation of her left thumb and development of mass	Excisional biopsy	NR
Sasso *et al.,* 2019^2^	11	Woman	Third of the extensor region of the left forearm above the ulnar diaphysis	NR	Asymptomatic nodular lesion in the left upper limb. Rapid growth	Surgical excision (excisional biopsy)	NR
Pegado *et al.,* 2015^3^	69	Woman	Anterior proximal aspect of the left forearm	Arteriovenous haemodialysis fistula	Mass next to the existing arteriovenous haemodialysis fistula	Ultrasound guided biopsy, no treatment	NR
Bass *et al.,* 2024^4^	69	Woman	Right upper extremity	Right humeral pathological fracture several weeks prior to presentation. Right plasma cell mastitis and recurrent right pleural effusion of uncertain aetiology 2 mo prior to presentation	Right upper extremity pain, swelling and functional decline	CT guided biopsy Type II IPEH ∗Septic shock related to a pre-existing pulmonary infection. Died in the ICU a week following her biopsy	NR
Sartore *et al.,* 2011^5^	Mean age of 54 y (range 42–74)	Woman Woman Woman Woman	Proximal phalanx Proximal phalanx Middle phalanx Palm	NR NR NR NR	Solitary, slightly tender, reddish–blue, and subcutaneous mass with a maximum diameter of 1.1 cm	Surgical excision	NR NR NR NR
Patai *et al.,* 2020^6^	26	Woman	2nd metacarpophalangeal joint, palmar side of the right hand	NR	Palpable mass, grew in size; tenderness to palpation	Marginal excision	NR
	30	Woman	Proximal phalanx of the left thumb	NR	Palpable mass, grew in size; tenderness to palpation; limited range of motion	Marginal excision	NR
	24	Man	1st metacarpophalangeal joint, palmar side of the left hand	NR	Palpable mass, grew in size; tenderness to palpation; limited range of motion	Marginal excision	NR
	36	Man	Distal phalanx of the right thumb	NR	Discomfort, palpable mass, tenderness	Marginal excision	NR
	71	Woman	Distal phalanx of the left ring finger, dorsoradial side	NR	Discomfort, palpable mass	Marginal excision	NR
	31	Man	Distal phalanx of the left ring finger, dorsal side	NR	Palpable mass, changing in size; no local tenderness	Marginal excision	NR
	61	Woman	Middle phalanx of the right ring finger, palmar side	NR	Discomfort, palpable mass, tenderness	Marginal excision	NR
Louie *et al.,* 2023^7^	38	Man	Interphalangeal (IP) joint of the right thumb, ulnar aspect	Hammer blow	Palpable mass, limited range of motion	Marginal excision	NR
Liné *et al.,* 2017^8^	2	Woman	Right axilla	NR	Pain subcutaneous tumour	Marginal excision	NR
Lee *et al.,* 2004^9^	68	Woman	Anterior aspect of the left shoulder	NR	Palpable mass	Surgical exploration, surgical biopsy	NR
Romano *et al.,* 2017^10^	71	Man	3rd finger of left hand	Two masses on the finger: diagnosis of IPEH for the greater mass and calcifying aponeurotic fibroma for the smaller lesion	Palpable mass	Surgical excision	NR
Velazquez *et al.,* 2008^11^	58	Woman	Humeral artery	NR	Asymptomatic pulsatile humeral artery mass	Surgical excision and replacement of the vascular segment by a segment of inverted cephalic vein	NR
Shah *et al.,* 2022^12^	37	Woman	Volar aspect of the right wrist	NR	Numbness and tingling in her wrist and thumb	Surgical excision	NR
Kuo *et al.,* 1976^13^	12 46	Man Woman	Finger Thumb	NR NR	Nodular mass Purplish nodule	NR NR	NR NR
Van Houdt *et al.,* 2024^14^	Late 70s	Woman	Right axilla	History of bilateral breast cancer treated with surgery, chemotherapy and radiotherapy	Slowly growing, large bulging mass in the right axilla	Surgical excision	NR
Lysyy *et al.,* 2010^15^	59	Woman	Dorsal aspect of the right forearm	NR	Palpable mass, grew in size, and slight swelling of the right hand	Surgical excision	NR
Ero *et al.,* 2007^16^	58	Woman	Proximal interphalangeal joint of the 4th finger in the left hand	The patient had dealt with different types of vegetables (including squash, okra) one day before she noticed the lesion	Small purplish swelling on the dorsal side of her left 4th finger. Tender swelling in her left hand	Surgical excision	NR
Ng *et al.,* 2020^17^	19	Man	Volar aspect of the proximal phalanx of the right little finger	NR	Gradually enlarging lump with associated numbness over the volar aspect of the proximal phalanx of his right little finger	Surgical excision (excision biopsy)	Recurrence within 2 mo
Lim *et al.,* 2011^18^	49	Woman	Left index finger	NR	Slow growing but tender mass in her left index finger associated with decreased sensation on the ulnar half of the finger pulp	Surgical excision (excision biopsy)	NR
Almarghoub *et al.,* 2020^19^	17	Woman	Volar aspect of the left 4th MCP	NR	Painful mass on the volar side of her left 4th MCP	Local excision	NR
Kitagawa *et al.,* 2012^20^	Median age of 31 y (range 22–66)	Man Man Man Woman Woman	All lesions were found in the digits: two thumbs, two ring fingers, and one index finger	NR NR NR NR NR	Painful mass	Surgical excision	NR
							NR NR NR NR
Clifford *et al.,* 2004^21^	34	Man	Posterior distal right arm	NR	Soreness and swelling in the posterior right arm	Surgical excision	NR
Pesce *et al.,* 2018^22^	61	Man	Thenar eminence of the right hand	NR	Mass at the thenar eminence of the right hand	Surgical excision	NR
Wang *et al.,* 2018^23^	55	Woman	Left palmar aspect between the 3rd and 4th metacarpals	Left hand contusion with ecchymosis 2 y previously	Protruding mass lesion on the left palm	Surgical excision	NR
	18	Man	Ulnar side of the left hand	Hand contusion injury caused by a fall 2 mo previously	Progressive protruding mass lesion on the ulnar side of the left hand with rapid enlargement	Surgical excision	NR
Lauder *et al.,* 2019^24^	18	Man	Volar 3rd web space	History of blunt trauma	Mass of the volar 3rd web space without associated neurovascular deficits	Surgical excision	NR
Mitchell *et al.,* 2021^25^	48	Man	Dorsoradial aspect of the proximal phalanx of the right index finger	NR	Right index finger mass.	Surgical excision	NR
Macias *et al.,* 2022^26^	28	Woman	Right upper limb	NR	Multiple nodules on the right upper limb	NR	NR
Pantanowitz *et al.,* 2008^27^	51 52	Man Man	Palm Finger	NR NR	Mass Mass	NR NR	NR NR
Inalöz *et al.,* 2001^28^	34	Woman	Right thumb	History of pyogenic granuloma in the pulp of the right thumb following a sharp penetrating needle injury	Nodule in the right thumb, at the site of a previously excised pyogenic granuloma. Recurrent IPEH	Surgical excision	NR
Chang *et al.,* 2012^29^	30	Woman	Ulnar aspect of her right forearm	Fusiform ulnar artery aneurysm with partial, non-occlusive thrombus Histopathology of her resected aneurysm compatible with IPEH	Pulsatile right forearm mass and numbness of her 4th and 5th fingers	Ulnar artery aneurysm resection and placement of a reverse saphenous vein interposition graft harvested from her right lower extremity	NR
Titchener *et al.,* 2015^30^	43	Woman	Distal to the carpal tunnel between the deep and superficial flexor tendons	NR	Paraesthesia and pain affecting her right hand corresponding to the distribution of the median nerve	Carpal tunnel decompression and excision biopsy	NR
Hashimoto *et al.,* 1983^31^	91 patients. Average age of 31 y (range 9 mo–78 y)	56% (51) women 44% (40) men	16 – finger 11 – forearm and elbow 6 – upper arm and shoulder 5 – hand and wrist	NR	Slowly growing tumefaction. Bluish–reddish discoloration of the overlying skin. 33% (30) tender or painful	Surgical excision	NR
Romaní *et al.,* 1997^32^	55	Woman	Palmar surface of her left hand	Mammary neoplasia diagnosis 4 y previously. Treated with mastectomy and lymphadenectomy. Onset of lymphedema of the left upper extremity 2 y after the procedure	Nodular red lesion on the middle aspect of her left palmar area	Surgical excision	NR
Corni *et al.,* 2014^33^	73	Man	Volar side of the proximal phalanx (P1) of the index finger	NR	Rapidly growing bluish mass on the volar aspect of the left index finger	Surgical excision	NR
García-Macías *et al.,* 1990^34^	22	Man	Posterior face of the lower 3rd of the left arm	History of injury to that area of the arm 2 mo previously	Well delimited lesion, slightly painful to the touch	Surgical excision	NR
Civatte *et al.,* 1986^35^	40 50	Woman Woman	Finger Right shoulder	NR NR	NR NR	Surgical excision Surgical excision	NR NR
Shah *et al.,* 2010^36^	32	Woman	Posteromedial aspect of her left elbow	NR	Ache over the lump, and down the ulnar border of her forearm. Change in the sensibility within the ulnar nerve territory of her left hand	Surgical excision	NR
Tokyol *et al.,* 2005^37^	7	Woman	Volar aspect of the metacarpophalangeal joint of her right thumb	NR	Painless bluish mass, grew in size; tenderness when holding a pencil to write	Surgical excision	NR
Katzman *et al.,* 1997^38^	31	Woman	Ulnar side of the carpal canal of the right hand	NR	Pain in the palm and small finger of the right hand. Numbness and paraesthesia in the right and small fingers	Surgical excision + tenosynovectomy	Recurrence 1 year later. New surgical excision
Anthony *et al.,* 2008^39^	15	Woman	Volar aspect of the proximal phalanx of the right middle finger	NR	Painful mass in the middle finger of her dominant hand	Excision biopsy	Recurrence within 4 wks. New surgical excision. The surgical margins contained residual tumour. New recurrence 3 wks later, treated with external beam radiation therapy using photons (41.4 Gy over 58 elapsed treatment days)
Pins *et al.,* 1993^40^	19	Man	Right shoulder	Acute strain injury to his right shoulder while lifting weights 1 wk previously	Enlarging mass in his rights shoulder	1. Fine needle aspiration & open incisional biopsy 2. Pre-operative embolisation Blood supply to the mass originated from the thyrocervical trunk along the transverse cervical artery 3. Bloc excision	NR
Acharya *et al.,* 1980^41^	34	Man	Hypothenar region of the right hand	Juvenile rheumatoid arthritis	Painful mass in the hypothenar region of his right hand.	Surgical excision	NR
	60	Woman	Dorsum of the middle phalanx of the left ring finger	The lesion had been excised 18 mo previously elsewhere, with no pathological examination performed	Recurrent bluish nontender mass on the dorsum of the middle phalanx of her left ring finger	Surgical excision, no further recurrence	NR
	12	Woman	Right thumb	She also had multiple haemangiomas over the metacarpophalangeal joint areas of the index and long fingers	Slightly painful mass on the right thumb	Surgical excision of the mass and haemangiomas	NR
Wehbé *et al.,* 1986^42^	55	Man	Distal phalanx of the right ring finger	NR	Swelling of the pulp of his right ring finger. The mass contained a speck of calcification and had eroded the distal phalanx	Surgical excision performed after surgical biopsy that had revealed only scar tissue	NR
Stewart *et al.,* 1994^43^	80	Woman	Face and neck and upper aspect of her arms	NR	Multiple red violaceous nodules distributed on her face and neck and upper aspect of her arms	Surgical excisions	NR
Busanelli *et al.,* 1988^44^	18	Man	Ulnar side of the middle phalanx of the 4th finger of the left hand	History of trauma followed by a swelling on the 4th finger of the left hand at the age of 7. Three surgical excisions, the first one with clinical diagnosis (no histological examination) of cyst and the other two with histological diagnosis of angioma	Recurrent swelling painful to palpation on the 4th finger of the left hand. Pain following hand use and minor trauma	Surgical excision	NR
Julliard *et al.,* 1983^45^	62	Man	Dorsal side of the wrist Outer side of the left 1st interphalangeal joint	NR	Two independent tumors on the left hand	Surgical excision	NR
Borrelli *et al.,* 1992^46^	23	Woman	Left hypothenar eminence	History of minor trauma to the hand. The patient was left handed	Small painful mass in her left hypothenar eminence	Surgical excision	NR
Gordon *et al.,* 1988^47^	62	Woman	Left forearm	Oesophageal webs, aspergilloma, and advanced sarcoidosis, for which she was receiving oral prednisone therapy (5 mg every other day)	Two flesh coloured painless nodules on her left forearm	Biopsy, no treatment	NR

CT = computed tomography; ICU = intensive care unit; NR = not reported; mo, months; wks, weeks; NR, not registered

∗ All references cited in this table correspond to the complete literature review and are additional to the references included in the main manuscript. The full list of the 47 included studies is provided in Appendix A.

Data from 103 patients were collected: 41 were women (39.8%), 24 were men (23.3%), and sex was not reported in 38 cases (36.9%). The mean age was 44 years (range 2 – 80 years).

The lesion was located in: the fingers in 49 patients (47.6%): ten in the thumb (20.4%), four in the index finger (8.2%), two in the middle finger (4.1%), ten in the ring finger (20.4%), one in the small finger (2.0%), and 22 were unspecified (44.9%); the hand and wrist in 18 cases (17.5%): eleven in the hand (61.1%), two in the wrist (11.1%), and five unspecified (27.8%). The lesion involved the forearm and elbow in 22 patients (21.4%): ten in the forearm (45.5%), one in the elbow (4.5%), and eleven unspecified (50.0%). The upper arm and shoulder were affected in 14 cases (13.6%): three in the upper arm (21.4%), five in the shoulder or axilla (35.7%), and six unspecified (42.9%).

Only one case (1.0%) was classified as type II IPEH, while all remaining cases (99.0%) were classified as type I, including this case.

Surgical excision of the mass was performed in 95 patients (92.2%). Three (2.9%) were not treated, follow up was not reported in two cases, and one patient died of unrelated comorbidities after the biopsy. Treatment was not reported in five cases (4.9%).

Among the surgically treated patients, specific procedures included: surgical excision and replacement of the vascular segment with an inverted cephalic vein segment (one patient, 1.1%), ulnar artery aneurysm resection and placement of a reverse saphenous vein interposition graft (one patient, 1.1%), carpal tunnel decompression (one patient, 1.1%), tenosynovectomy (one patient, 1.1%), and pre-operative embolisation (one patient, 1.1%).

Recurrence was reported in three patients (2.9%) and treated with repeat surgical excision. A second recurrence occurred in one patient due to residual tumour at the margins, and adjuvant radiotherapy was administered.

## Discussion

IPEH is a rare vascular entity that can arise in any blood vessel and accounts for approximately 2% of skin tumours.^5^ It typically involves subcutaneous tissue and rarely affects the superficial skin layers. Commonly found in areas exposed to trauma, the head, neck, and upper extremities are the most frequent locations.^5^ The condition shows a slight predominance in women and most frequently occurs during the third and fourth decades of life.^5^ Its aetiology remains uncertain and is probably multifactorial. The most widely accepted hypothesis suggests a reactive process triggered by thrombotic or inflammatory stimuli within the vessel wall, where recanalisation following thrombosis induces excessive endothelial proliferation.^1^^,^^6^^,^^7^ Trauma, hormonal influence, altered growth factor expression, pre-existing vascular abnormalities, and thrombosis have been implicated;^6^^,^^7^ however, no precipitating factor can be identified in up to 70% of cases, as in the present case.^5^

Clinically, IPEH typically presents as a slow growing, firm, non-pulsatile subcutaneous nodule, often with a red–blue discoloration related to intravascular dilation and obstruction. It may mimic other benign or malignant soft tissue lesions, including angiomas, lipomas, vascular malformations, cysts, and sarcomas.^8^

Initial evaluation relies on non-invasive imaging, although definitive diagnosis requires histopathological confirmation. On ultrasound, IPEH usually appears as a well defined, hypoechoic, lobulated mass. Magnetic resonance imaging often demonstrates a well circumscribed lesion and shows an association with a dominant vessel in approximately 73% of cases.^5^^,^^8^

Histopathological examination remains the gold standard for diagnosis, as clinical and radiological features are non-specific. The most important differential diagnosis is angiosarcoma, from which IPEH is distinguished by the absence of necrosis, significant cytological atypia, infiltrative growth, and high mitotic activity.^4^

Given its benign nature, management depends on lesion location and symptomatology. Complete surgical excision with clear margins is the treatment of choice and is associated with an excellent prognosis and low recurrence rates. Routine follow up is generally not required after complete excision; however, it may be considered in selected cases, particularly in the setting of incomplete resection or secondary forms of IPEH, which are associated with a higher risk of recurrence.^9^

In conclusion, IPEH is a rare benign vascular lesion that may clinically and radiologically mimic malignant vascular tumours, making pre-operative diagnosis challenging. Although features such as well defined margins and slow growth may suggest benignity, these finding are insufficiently reliable to exclude malignancy. Consequently, complete surgical resection remains the standard initial approach, providing both definitive diagnosis and treatment. The key clinical implication is not to avoid surgery, but to avoid unnecessarily aggressive oncological resections when pre-operative and intra-operative findings are more consistent with a benign vascular lesion. Awareness of IPEH may therefore support a more conservative surgical strategy without compromising complete excision, which offers an excellent prognosis.

## Use of generative AI tools

During the preparation of this work the authors used ChatGPT (OpenAI) to assist with English language translation and manuscript drafting. After using this tool, the authors reviewed and edited the content as needed and take full responsibility for the content of the publication.

## Conflict of interest and funding

The authors declare that they have no conflict of interest. This research did not receive any specific grant from funding agencies in the public, commercial, or not-for-profit sectors.

## Appendix A Studies included in the literature review (*n* = 47)

1. Hutcheson EL, Picarella EA, Blevins PK. Masson's tumor of the hand: a case report and brief literature review. Ann Plast Surg. 2012;69:338–339. PMID: 21921791.
2. Sasso SE, Naspolini AP, Milanez TB, Suchar G. Masson's tumor (intravascular papillary endothelial hyperplasia). An Bras Dermatol. 2019;94:620–621. PMID: 31777368; PMCID: PMC6857574.
3. Pegado PF, Ordi QC, Roche S, Rivas AG, Domiguez RO. Intravascular papillary endothelial hyperplasia (Masson tumor) mimicking a sarcoma and developing from an arteriovenous hemodialysis fistula. Skeletal Radiol. 2015;44:859–862. PMID: 25427786.
4. Bass R, Siegal G, Kotha A, Melenevksy Y. Soft tissue hemangioma of the right upper extremity with intraosseous extension and secondary intravascular papillary endothelial hyperplasia. Skeletal Radiol. 2024. PMID: 38995413.
5. Sartore L, Voltan A, Tomat V, Bassetto F, Salmaso R. Masson's disease in hand surgery: a clinicopathologic study of four cases. J Hand Surg Eur Vol. 2011;36:694–697. PMID: 21636622.
6. Patai BB, Peterfy N, Szakacs N, Sapi Z, Hetthessy JR. Papillary endothelial hyperplasia (Masson tumor) of the hand. Pathol Oncol Res. 2020;26:2083–2090. PMID: 32671676; PMCID: PMC7471200.
7. Louie D, Elahi L, Balagué N, Bouvet C. A case of Masson tumor within an arteriovenous malformation of the thumb. Hand Surg Rehabil. 2023;42:170–171. PMID: 34757236.
8. Liné A, Sanchez J, Jayyosi L, Birembaut P, Ohl X, Poli-Mérol ML, François C. Papillary endothelial hyperplasia (Masson's tumor) in children. Ann Chir Plast Esthet. 2017;62:232–237. PMID: 27346753.
9. Lee SH, Suh JS, Lim BI, et al. Intravascular papillary endothelial hyperplasia of the extremities: MR findings with pathologic correlation. Eur Radiol. 2004;14:822–826.
10. Romano N, Fischetti A, Mussetto I, Bianchi R, Muda A. Masson's tumor and calcifying aponeurotic fibroma: two rare soft tissue lesions in the same finger. Med Ultrason. 2017;19:457–461. PMID: 29197926.
11. Velázquez CJ, Font JI, Torres F, Araji O, Barquero JM. Masson tumor as humeral artery aneurysm. Ann Vasc Surg. 2008;22:127–129. PMID: 18083332.
12. Shah VJ, Sung K, Goodwill V, Huang BK, Abrams R. Intravascular papillary hemangioendothelioma disguised as a peripheral sheath tumor of median nerve at the wrist: a case report and literature review. Skeletal Radiol. 2023;52:1421–1426. PMID: 36513787; PMCID: PMC10188408.
13. Kuo T, Sayers CP, Rosai J. Masson's vegetant intravascular hemangioendothelioma: a lesion often mistaken for angiosarcoma. Cancer. 1976;38:1227–1236. PMID: 986234.
14. Van Houdt M, Fidlers T, Neven P, Han S. (Intravascular) papillary endothelial hyperplasia or Masson's tumour of the axilla in a patient with a history of breast cancer. BMJ Case Rep. 2024;17:e261765. PMID: 39532336.
15. Lysyy O, Schwartz I, Kolander Y, Strauss S. Sonographic features of intravascular papillary endothelial hyperplasia (Masson's tumor) in the forearm. J Clin Ultrasound. 2011;39:301–303. PMID: 21547933.
16. Erol O, Ozçakar L, Uygur F, Keçik A, Ozkaya O. Intravascular papillary endothelial hyperplasia in the finger. J Cutan Pathol. 2007;34:806–807. PMID: 17880590.
17. Ng HJH, Sio BR, Desai V, Chew KM, Rajaratnam V. Intravascular papillary endothelial hyperplasia: case report of a recurrent Masson's tumor of the finger and review of literature. J Hand Microsurg. 2021;13:164–168. PMID: 34602798; PMCID: PMC8463135.
18. Lim R, Tay SC, Yam A. Intravascular papillary endothelial hyperplasia of the finger presenting as a digital nerve schwannoma. J Hand Surg Eur Vol. 2011;36:612–613. PMID: 21730005.
19. Almarghoub MA, Shah Mardan QNM, Alotaibi AS, Ahmed NK, Alqahtani MS. Masson's tumor involving the hand. Int J Surg Case Rep. 2020;70:223–226. PMID: 32422583; PMCID: PMC7229427.
20. Kitagawa Y, Tamai K, Kim Y, Hayashi M, Makino A, Takai S. Intravascular papillary endothelial hyperplasia of the digit: MRI features with histological correlation. J Hand Surg Eur Vol. 2013;38:306–312. PMID: 22759484.
21. Clifford PD, Temple HT, Jorda M, Marecos E. Intravascular papillary endothelial hyperplasia presenting as a triceps mass. Skeletal Radiol. 2004;33:421–425. PMID: 15205930.
22. Pesce V, Bizzoca D, Notarnicola A, et al. Intravascular papillary endothelial hyperplasia of the hand mimicking a hemangiopericytoma. SAGE Open Med Case Rep. 2018;6:2050313X17752851. PMID: 29348916; PMCID: PMC5768252.
23. Wang CY, Hong CC, Huang ZY, Wang CH. Intravascular papillary endothelial hyperplasia after hand contusion injury. Acta Orthop Traumatol Turc. 2019;53:154–156. PMID: 30292474; PMCID: PMC6506812.
24. Lauder A, Bentley RC, Mithani SK. Intraneural Masson tumor of the hand. Eplasty. 2019;19:ic9. PMID: 30858903; PMCID: PMC6402412.
25. Mitchell M, Riccio C, Mukit M, et al. Masson's tumor of the finger. Eplasty. 2021;21:ic5. PMID: 34025903; PMCID: PMC8120262.
26. Macias LL, Dos Santos LM, de Morais PM, et al. Multiple nodules on the unilateral upper limb. JAAD Case Rep. 2022;29:59–61. PMID: 36193245; PMCID: PMC9525730.
27. Pantanowitz L, Duke WH. Intravascular lesions of the hand. Diagn Pathol. 2008;3:24. PMID: 18513426; PMCID: PMC2426672.
28. Inalöz HS, Patel G, Knight AG. Recurrent intravascular papillary endothelial hyperplasia developing from a pyogenic granuloma. J Eur Acad Dermatol Venereol. 2001;15:156–158. PMID: 11495526.
29. Chang K, Barlaben A, Farley S. Masson's tumor in the ulnar artery. J Vasc Surg. 2012;56:223–225. PMID: 22387264.
30. Titchener AG, Arenas-Prat J. Carpal tunnel syndrome secondary to Masson's tumour. J Clin Orthop Trauma. 2015;6:281–282. PMID: 26566345; PMCID: PMC4600886.
31. Hashimoto H, Daimaru Y, Enjoji M. Intravascular papillary endothelial hyperplasia. Am J Dermatopathol. 1983;5:539–546. PMID: 6666836.
32. Romaní J, Puig L, Costa I, de Moragas JM. Masson's IPEH mimicking Stewart-Treves syndrome. Cutis. 1997;59:148–150. PMID: 9071555.
33. Corni V, Lucchina S, Fusetti C. Is Masson's tumour only a slow-growing benign neoplasm? Hand Surg. 2014;19:445–447. PMID: 25288292.
34. García-Macías MC, Abad M, Alonso MJ, Flores T, Bullón A. Masson's vegetant intravascular hemangioendothelioma. Acta Cytol. 1990;34:175–178. PMID: 2108526.
35. Civatte J, Castro G, Chemaly P, Fois C. Hemangioendothelioma vegetante intravascular. Med Cutan Ibero Lat Am. 1986;14:421–424. PMID: 3550318.
36. Shah AK, Grant I. Intravascular papillary endothelial hyperplasia causing a soft-tissue mass in the upper limb. Hand Surg. 2010;15:31–33. PMID: 20422724.
37. Tokyol C, Demir Y, Aktepe F. Cutaneous IPEH of the hand. Turk J Pediatr. 2005;47:71–74. PMID: 15884634.
38. Katzman B, Caligiuri DA, Klein DM, et al. Recurrent intravascular papillary endothelial hyperplasia. J Hand Surg Br. 1997;22:113–115. PMID: 9061543.
39. Anthony SG, Mudgal CS, DeLaney TF, et al. Recurrent IPEH treated with radiation therapy. J Bone Joint Surg Br. 2008;90:95–97. PMID: 18160508.
40. Pins MR, Rosenthal DI, Springfield DS, Rosenberg AE. Florid extravascular papillary endothelial hyperplasia. Arch Pathol Lab Med. 1993;117:259–263. PMID: 8442671.
41. Acharya G, Merritt WH, Theogaraj SD. Hemangioendotheliomas of the hand. J Hand Surg Am. 1980;5:181–182. PMID: 7358960.
42. Wehbé MA, Otto NR. Intravascular papillary endothelial hyperplasia in the hand. J Hand Surg Am. 1986;11:275–279. PMID: 3958464.
43. Stewart M, Smoller BR. Multiple lesions of IPEH. Arch Pathol Lab Med. 1994;118:315–316. PMID: 8135640.
44. Busanelli T, Tampieri PF, Mingazzini T. Masson hemangioendothelioma plurirecurrent. Chir Organi Mov. 1988;73:269–272. PMID: 3243112.
45. Julliard FA, Baviera EE. Masson lesion simulating angiosarcoma. Ann Chir Main. 1983;2:59–61. PMID: 9382637.
46. Borrelli L, Ciniglio M, Maffulli N, Del Torto M. IPEH in a fencer’s hand. Pathologica. 1992;84:551–556. PMID: 1491898.
47. Gordon ML, Kest E, Phelps RG. Forearm nodule. Arch Dermatol. 1988;124:263–264. https://doi.org/10.1001/archderm.1988.01670020081023
